# Importance of Endogenous Fibrinolysis in Platelet Thrombus Formation

**DOI:** 10.3390/ijms18091850

**Published:** 2017-08-25

**Authors:** Ying X. Gue, Diana A. Gorog

**Affiliations:** 1 Department of Cardiology, East and North Hertfordshire NHS Trust, Hertfordshire SG1 4AB, UK; y.gue@nhs.net; 2 Department of Postgraduate Medicine, University of Hertfordshire, Hertfordshire AL10 9AB, UK; 3 National Heart & Lung Institute, Imperial College, London SW3 6LY, UK

**Keywords:** endogenous, spontaneous, thrombolysis, fibrinolysis, thrombosis, platelets, cardiovascular

## Abstract

The processes of thrombosis and coagulation are finely regulated by endogenous fibrinolysis maintaining healthy equilibrium. When the balance is altered in favour of platelet activation and/or coagulation, or if endogenous fibrinolysis becomes less efficient, pathological thrombosis can occur. Arterial thrombosis remains a major cause of morbidity and mortality in the world despite advances in medical therapies. The role endogenous fibrinolysis in the pathogenesis of arterial thrombosis has gained increasing attention in recent years as it presents novel ways to prevent and treat existing diseases. In this review article, we discuss the role of endogenous fibrinolysis in platelet thrombus formation, methods of measurement of fibrinolytic activity, its role in predicting cardiovascular diseases and clinical outcomes and future directions.

## 1. Background

### 1.1. Haemostasis

Haemostasis is a complex sequence of biochemical response to injury to allow formation of a blood clot and repair of damaged endothelium. The maintenance of the equilibrium between coagulation and fibrinolysis is vital, as imbalance would lead to abnormal bleeding or increased risk of thrombosis. Thrombosis is pathological clot formation within the blood vessels in the absence of injury. In fact, the recognition of thrombosis has shaped treatment and prevention of vascular events such as acute coronary syndrome (ACS), which is typically caused by arterial thrombosis following the rupture of vulnerable atheroma within the arterial wall [1,2,3].

### 1.2. Atherosclerosis and Plaque Rupture

Atherosclerosis is induced by many factors including endothelial dysfunction, elevated low-density lipoprotein (LDL), oxygen free radicals and hypertension. These factors trigger an inflammatory response within the endothelial cells and, with repeated insults, lead to proliferation of the smooth muscle cells and formation of lipid-rich or fibrous plaques which intrude into the lumen and alter blood flow dynamics within [4]. Plaques have varying morphology and propensity to rupture [5]. Rupture of the plaque allows the contents of the plaque and the subendothelium to come into contact with the contents of flowing blood. Emphasis has been placed on thin-cap fibroatheroma (TCFA) as they are considered very vulnerable to rupture and are most causally associated with the occurrence of coronary thrombosis [6].

### 1.3. Platelet Aggregation and Thrombus Formation

Following plaque rupture, the exposure of tissue factor and collagen creates a pro-thrombotic environment by initiating the coagulation cascade, platelet aggregation and thrombus formation [1]. Von Willebrand factor (vWF), platelet glycoprotein receptors, adenosine diphosphate (ADP), thromboxane A_2_ (TxA2) and thrombin all play important roles in platelet activation and recruitment of other platelets [7]. Thrombin is also responsible for converting fibrinogen into fibrin, which stabilizes the platelet-platelet contacts leading to thrombus formation.

## 2. Regulators of Endogenous Fibrinolysis

### 2.1. Endogenous Fibrinolysis

If thrombus can propagate indefinitely, it will lead to complete occlusion of the vessel and loss of blood flow resulting in disastrous tissue damage, as in the case of acute myocardial infarction or cerebrovascular events or acute peripheral vascular occlusion [8]. Endogenous or spontaneous fibrinolysis is the physiological counter-measure against lasting arterial thrombosis. It is divided into two key steps: (1) activation of plasminogen to serine proteinase plasmin by tissue (tPA) and urokinase (uPA) plasminogen activator; and (2) breaking down of fibrin into fibrin degradation products, thereby dissolving the thrombus to allow restoration of blood flow [9].

### 2.2. Regulation of Fibrinolysis

Hyperfibrinolysis can result in uncontrolled bleeding, as in the case with disseminated intravascular coagulation (DIC) where systemic inflammation causes increased consumption of fibrin and clotting factors. In a healthy individual, fibrinolysis is regulated by inhibiting plasminogen activator or antagonizing plasmin through α-2-antiplasmin. Plasminogen activator inhibitor (PAI) has been found to work on both tPA and uPA [10,11]. Thrombin-activatable fibrinolysis inhibitor (TAFI) is a glycoprotein which works by reducing activation of plasminogen [10]. The structure of the thrombus also impact on its lysis [12,13] and Factor XIII (FXIII) plays an important role [14]. FXIII not only helps in formation of the fibrin network, but also crosslinks α-2-antiplasmin to fibrin, making it more resistant to fibrinolysis [15]. Lipoprotein (a) (Lp(a)) is a subclass of low density lipoprotein which reduces fibrinolysis through competitive inhibition of plasmin [16].

Plasminogen activator inhibitors are the main glycoproteins responsible for inhibiting the actions of tPA and uPA, with PAI-1 being the most relevant as it is produced by platelets and endothelial cells. PAI-2 is produced by the placenta and is only present in detectable amount during pregnancy. Upon stimulation by thrombin, PAI-1 is released from within platelets as a protective mechanism against premature lysis [11]. PAI-1, synthesized in platelets and endothelial cells, binds with tPA and uPA in a 1:1 ratio to form a stable compound which is then cleared by the liver [11,17].

Thrombin-activatable fibrinolysis inhibitor is a fibrinolysis inhibitor that is converted into its active form, TAFIa, during coagulation after thrombin cleavage. It acts by reducing plasminogen binding to fibrin leading to increase in lysis time [10,18,19]. Its generation is dependent on thrombin levels and greatly potentiated by thrombomodulin [19,20].

α-2-antiplasmin is a serine protease inhibitor which is produced in the liver. It inhibits fibrinolysis via three mechanisms: (1) forming a complex with plasmin; (2) inhibits adsorption of plasminogen onto fibrin; and (3) cross-links with FXIIIa to make fibrin more resistant to plasmin [8].

Factor XIII is a clotting factor which is activated by thrombin in the presence of calcium. Its role is in generating cross-links between fibrin strands within fibrin mesh which strengthens it. It also cross-links inhibitors of fibrinolysis, such as α-2-antiplasmin and TAFI, onto fibrin reducing its solubility and making it more resistant to the effects of plasmin [15]. FXIII is present in large quantities within platelets thereby making platelet rich clots more resistant to lysis than whole blood clots [21].

Lipoprotein (a) competitively binds to fibrin as its molecular structure is similar to plasminogen, leading to an anti-fibrinolytic effect [16]. It also increases synthesis of PAI-1 by endothelial cells which further reduces plasmin levels [16]. Lp(a) is also believed to be involved in atherogenesis and studies have shown links between Lp(a) levels and cardiovascular risk [16,22,23,24]. A summary of the regulators of fibrinolysis is provided in Table 1.

## 3. Measurement of Endogenous Fibrinolysis

### 3.1. Clinical Tests

Electrocardiography (ECG) is the gold-standard non-invasive test to evaluate chest pain. Its use has directed classification of ACS and its treatment. The presence of ST-segment elevation on an ECG is usually an indication of acute thrombotic occlusion of the coronary artery. The natural history of ECG changes in ST-segment elevation myocardial infarction (STEMI) begins with hyperacute T-waves, ST-segment elevation, T-wave inversion, ST-segment resolution followed lastly by abnormal Q waves which indicates transmural infarction. Although in almost all patients with STEMI eventually the ST-segment elevation will eventually resolve with time within 12 h, even if reperfusion does not occur, early spontaneous resolution is associated with improved prognosis [25,26]. It is a crude measure of reperfusion and therefore a marker of fibrinolysis (spontaneous or iatrogenic). Serial ECGs can be used in the setting of STEMI to measure the time taken for reperfusion to occur, which is a surrogate measure of fibrinolytic activity. Therefore, patients with delayed ST-segment recovery are considered to be at higher risk and may require further treatment and closer follow up.

Angiography is the use of X-rays and contrast agent to assess blood vessels. In the realm of cardiology, coronary angiography, either by direct fluoroscopy or using computed tomography, is used to assess coronary artery disease. It can identify luminal narrowing within the coronary arteries and flow distal to the disease. Thrombolysis In Myocardial Infarction (TIMI) flow grade is widely used to classify epicardial blood flow [27]. The presence of TIMI 3 flow (normal flow filling distal coronary completely) is associated with improved outcomes compared to lower grades [28]. The restoration of flow on angiography is an indication that fibrinolysis has resulted in the elimination of thrombotic occlusion. Angiography is currently the “gold-standard” measure of the restoration of blood flow in the epicardial coronary arteries in patients with coronary artery disease.

### 3.2. Laboratory Measurement of Fibrinolysis

Activity of fibrinolytic pathways can be measured through various factorial assays and biomarkers such as tPA [29,30,31], PAI-1 [32,33], α-2-antiplasmin and plasma-α-2-antiplasmin (PAP) complex [32,34], fibrin degradation products (d-dimer and soluble fibrin) [29,32,34,35], TAFI [32,36] and LP(a) [32,33,37]. Although the measurement of these markers reflects the activity of fibrinolytic pathway, their clinical use and predictive values remains limited [32]. It is very difficult, with so many individual components of the pathway, to build up a complete or comprehensive overview of the efficiency of the fibrinolytic pathways. Furthermore, the relative contribution of different individual components may vary in different clinical scenarios.

As the structure of the fibrin network regulates mechanical stability and its resistance to fibrinolysis, the assessment of clot structure can provide information about thromboembolic risk and susceptibility to lysis [38]. Fibrin clot permeability is the measure of how tightly packed the fibrin clot is and has been identified to be altered in patients with coronary artery disease (CAD), diabetes and relatives of family with premature CAD [12,39,40,41]. The method of performing this requires the use of citrated plasma mixed with thrombin to allow clot formation. Volume of buffer flowing through the gel is then added into a formula to calculate the permeation coefficient (*K*s) [42]. The main limitation of this assessment is that the range of values is large, thereby making inter-laboratory comparison difficult even with standardisation of measurements [43].

### 3.3. Clinically Applicable Tests of Fibrinolysis

Thromboelastography or TEG^®^ and rotational thromboelastometry or ROTEM^®^ are point of care, global test of coagulation status, simultaneously assessing clot development, stabilization, and dissolution. It utilizes a pin suspended by a torsion wire into a cylinder or an optical detector in the case of ROTEM^®^, to measure the physical properties of a clot. As blood clot formation occurs around the pin, fibrin strands form between the cylindrical cup and pin. The rotation of the cylindrical cup will be transmitted to the pin whose displacement is then picked up by the torsion wire. This is analysed and presented in graphical form by the machine to allow analysis of different stages of coagulation and fibrinolysis [44]. It is able to provide clot formation time and rate, maximum amplitude (MA) or clot strength and clot lysis time (CLT). It was designed to be used with native blood but modification with different activators and inhibitors have been used [45,46,47] although the correlation has been poor [48]. Its main use remains in assessing perioperative risks of bleeding, with questionable use with regards to assessment of fibrinolytic status [49]. Its main limitation in assessing fibrinolysis is that it employs a low-flow, static-type situation that more closely resembles venous, rather than arterial thrombosis.

The Global Thrombosis Test (GTT, Thromboquest Ltd., London, UK) is a relatively new automated, point-of-care test that simultaneously assesses platelet reactivity, thrombosis, and fibrinolytic activity, from a native whole blood sample [50]. Blood passing through a plastic conical tube with narrow gaps generates high shear stress. This mimics flow within a narrowed vessel, activates platelets and induces thrombus formation. Reduction of flow, as detected by an optical sensor, is expressed as occlusion time (OT). Blood flow resumes following spontaneous fibrinolysis, and the time taken to do so is expressed as the lysis time (LT). The GTT provides a more comprehensive test to evaluate thrombosis and lysis under physiological conditions that closely resemble arterial thrombosis models. The evaluation of GTT results in association with clinical outcomes has shown promise in various clinical settings for identifying patients at risk of future adverse cardiovascular event [51,52].

### 3.4. Measuring Lysis and Its Clinical Impact

Cardiovascular disease (CVD) is the leading cause of death globally, accounting for 32% of deaths [53]. There are many interventions in place to modify risk factors for patients at a population level. With the appreciation of the role that thrombosis and fibrinolysis plays in CVD [54], could we equate the measurement of fibrinolysis into defining specific high risk patient groups which could lead to targeted preventative therapy?

Prediction of clinical outcome, or clinical risk of future adverse events, is a vital process of management of patients with pathological conditions. It allows clinicians to decide the need for more aggressive treatments which come with a price of increased likelihood of side effects. Scoring systems have been used in several areas of clinical medicine and normally take into account parameters which can affect clinical outcome. The use of the Global Registry of Acute Coronary Events (GRACE) score to risk stratify patients with ACS and the QRisk2 score used in the UK are examples [55,56]. The use of the GRACE score has allowed hospitals to identify patients who are higher risk for whom in-patient management and earlier intervention is warranted [57,58]. The QRisk2 score allows the physician to incorporate simple demographics and blood tests to provide patients with their individual risk of a cardiovascular event expressed as a percentage over 10 years [56].

Although ECG is a low cost, low risk and easily available test, its use in screening of asymptomatic, low risk population is negligible [59]. In patients with conventional risk factors for CVD, the use of screening ECG does not add any value to risk management [59]. Early resolution of ST segment elevation on the ECG of patients with ACS that have undergone thrombolysis has been shown to be associated with improved prognosis [25]. In the substudy of HORIZONS-AMI trial, absence of ST-segment resolution post primary percutaneous coronary intervention (PPCI) has been found to be a predictor of major adverse cardiovascular events (MACE) and the need for recurrent target vessel revascularisation [60]. This has also been shown in other trials [61,62]. Lønborg J. et al. [63] evaluated spontaneous ST-segment resolution and identified improved long-term outcome in patients with resolved ST-segment deviation prior to PPCI. Resolution of ST-depression in patients with STEMI by ≥50% is associated with more favourable outcomes [64].

With angiography being the standard measure of localised epicardial fibrinolytic activity in patients with STEMI, its usefulness in guiding prognosis is well documented. Early infarct-related artery patency at angiography, an indicator of spontaneous fibrinolysis, is an independent predictor of lower 1 year mortality [65], higher TIMI flow post percutaneous coronary intervention (PCI), more rapid ST-segment resolution and favourable clinical outcome after 90 days [66]. The presence of TIMI 3 flow post PPCI is associated with better outcome [67] and conversely TIMI flow ≤2 is associated with adverse events [68].

The use of coronary computed tomography angiography (CCTA) and coronary calcium scoring for detection of coronary artery disease is increasing. There have been various studies which involved the use of CCTA to screen for coronary artery disease. The Subclinical COronary Atheroscleorosis Updated With Coronary cT Angiography (SCOUT) study enrolled 1000 asymptomatic individuals of different risk groups and identified plaques in 22% of the patients [69]. However, the prognosis of these patients was good and therefore the risk versus benefit of using screening CCTA remains questionable. A randomised trial looked into screening of high risk diabetics using CCTA (FACTOR-64) did not show evidence to support the use of CCTA screening [70].

Biomarkers, including factorial assays of fibrinolysis pathways have been used with varying degrees of success in identifying high risk cohorts, however their overall usefulness remains controversial and not helpful in individual patients [32]. There has been weak correlation shown between tPA and the development of coronary disease [29,71] however this is not supported other studies [72]. PAI-1 and its relation to cardiovascular events are in similar predicament [72,73]. In predicting outcomes, PAI-1 has been found to be independent predictor of in-hospital and one year mortality [74] and MACE following PCI [33,75]. It has also shown association with higher all cause mortality and ACS [76]. Measurement of TAFI level has had conflicting results with regards to predicting CAD risk with some studies showing a low level being cardioprotective [77,78] whilst others stating the opposite [79,80]. High levels of TAFI levels were shown to predict stent restenosis [81]. A recently published large study found that elevated LP(a) levels were associated with an increased risk of major adverse cardiovascular events (MACE) and CVD especially in diabetic populations [82], whereas previous studies have shown weak correlations [37,83]. However, Lp(a) has shown no associations with mortality in patients with established coronary artery disease [84] or MACE post PCI [33].

Although there are prospects for the use of biomarkers in risk detection of populations at risk, limitations still exist. The limitations stem from the complex nature of fibrinolysis. Although the measurement of different assays may reflect part of the activity, the complex interaction between the structure of the thrombin clot, cells within the arterial blood i.e., neutrophils and effects of drugs added into the mix limits its predictive value [32]. Furthermore, levels of these factorial components may fluctuate over time and in response to multiple clinical factors, so prediction based on an isolated sample has very limited value.

Clot permeability and the assessment of fibrin clot properties are more useful in prediction of venous thromboembolism [14,85,86]. Clots resistant to fibrinolysis have also been identified in patients with end-stage renal disease (ESRD) [87]. In patients with a family history of premature coronary artery disease, fibrin clots are thicker and less permeable [39]. Patients with type 2 diabetes have also been found to have lower permeability clots that are resistant to lysis [88,89]. In a matched case control study, patients with in-stent stenosis were found to have denser clots which were more resistant to lysis [90] and in patients with ischemic strokes, it has been found that less porous clots are associated with neurological deficit [91]. There have been very limited clinical studies exploring the relationship of clot properties with clinical outcome.

TEG^®^ and ROTEM^®^ have been in use for a long time in the prediction of bleeding and the requirement for blood and blood products in the settings of trauma resuscitation and in operating theatres [92,93,94,95]. Although their use in the capacity of hyperfibrinolysis is well described in the literature, with regards of hypofibrinolysis (namely thrombosis risk assessment), it has had less success [96]. High platelet-fibrin clot strength (MA) has been linked to increased ischemic events [97,98,99,100], the use of TEG^®^ in the detection of patients at CVD risk is extremely limited. A study comparing patients with different levels of angina looked at time to clot and clot strength using TEG^®^ and has shown a stepwise increase in clot strength from clinically stable to unstable stages of angina [101]. This might be an area where TEG^®^ may provide some insight.

Clinical studies evaluating the Global Thrombosis Test have shown relation between lysis time (LT), a measure of endogenous fibrinolysis, and MACE. A study of 300 patients with ACS revealed that LT >3000 s was identified as the optimal cut-point to predict MACE [51]. Even with adjustments for baseline cardiovascular risk factors, LT remained a statistically significant predictor of recurrent future MACE. In patients undergoing PPCI, pre-PPCI impaired endogenous fibrinolysis is associated with MACE whilst intact fibrinolysis was associated with spontaneous reperfusion, ST resolution and TIMI 3 flow pre-PPCI [102]. There have been clinical studies evaluating the use of the Global Thrombosis Test in the context of cardiovascular risk factors such as smoking and metabolic syndrome [103,104,105] and they have shown correlation between differences in LT and presence of risk factor. The GTT shows promise in the use of this technique for risk factor detection as well as assessing the impact of particular risk factors on thrombotic status.

The methods of measuring fibrinolysis and their clinical applications are summarised in Table 2.

## 4. Current Situation, Limitations and Future Directions

Cardiovascular disease remains the leading cause of mortality and morbidity in the world despite many prevention and treatment strategies. Currently in the UK, the screening for cardiovascular disease is done thorough risk scores (such as QRisk2) [56] to allow primary care practitioners to start primary prevention treatment for patients to avoid future thrombotic events. Traditional cardiovascular risk factors such as smoking, hypertension and hypercholesterolemia are the targets for primary prevention drugs.

Current treatment for arterial thrombosis relies on antiplatelet agents, such as aspirin, and P2Y_12_ inhibitors, such as clopidogrel or ticagrelor, to help alleviate the pro-thrombotic environment. However, use of such medications to reduce thrombosis in allcomers puts many patients at unnecessary risk of bleeding [106,107].

Although the use of fibrinolytic therapy is still recommended for STEMI if PPCI cannot be provided within 120 min [108], its use remains limited outside of it. With the understanding of the importance of spontaneous, endogenous fibrinolysis and its role in modulating platelet thrombus formation, medication that impacts favourably on this pathway may be an alternative approach to reperfusion or to regulate thrombus formation. This could be in the form of inhibition of regulators of fibrinolysis (TAFI inhibitors [109,110], PAI-1 inhibitors [111,112] and α-2 plasmin inhibitors [113,114]).

The use of clinical tests such as ECG is incorporated as a baseline assessment of chest pain to allow classification of pathology i.e. STEMI, NSTEMI, unstable angina which is useful in specific treatment pathways to be followed. Angiography with PCI is the main treatment for patients presenting with ACS. These clinical tests will likely remain as the backbone for assessment of CVD.

Laboratory assays of individual components of the fibrinolytic pathway may be used as a research tool in population studies, but plays little role in the clinical setting in individual patients as the use of such factorial assays to determine risk and clinical outcome has been controversial. Although Lp(a) has shown promise in identifying high risk patients with diabetes [82], there is still work to be done for it to have a place in risk stratification for CVD. The lack of standardised approach to fibrin clot structure assessment and permeability studies limits reproducibility and usefulness [43]. However, the possibility of an automated system might allow this technique to be used more widely [115].

Point-of-care global assays have a more important role within the clinical environment. Thromboelastography, including TEG^®^ and ROTEM^®^, has become well established in the assessment hyperfibrinolysis, namely bleeding, in emergency and theatre settings but has not been shown to help predict recurrent thrombotic events due to hypofibrinolysis. Standardisation techniques are also required to allow for universal interpretation of results [116]. The Global Thrombosis Test provides a more physiological assessment of both thrombus formation and thrombus lysis in a high shear setting reflective of arterial flow conditions. Studies suggest that it may play a role in identifying patients at high risk of future cardiovascular events and predict outcomes in patients following cardiovascular events.

## 5. Conclusions

Endogenous fibrinolysis plays an important role in impeding the continuous propagation of a growing platelet thrombus. It is an expanding area of interest in cardiovascular research and provides an alternative viewpoint to the approach of treatment of cardiovascular disease. A number of different ways of assessing the capacity of the fibrinolytic pathways are available, which may aid future cardiovascular risk stratification and the tailoring of medications to favourably alter this.

## Figures and Tables

**Table 1:** Regulators of fibrinolysis and their role.

Regulators of Fibrinolysis	Role
PAI-1	Inhibits tPA/uPA
TAFI	Decreases binding of plasminogen to fibrin
α-2-antiplasmin	Forms complex with plasmin Increase adsorption of plasminogen on fibrin Crosslinks FXIIIa
FXIII	Generates crosslinks between fibrin strands Crosslinks other inhibitors (TAFI, α-2-antiplasmin) onto fibrin
Lp(a)	Competitively binds to plasminogen Increases synthesis of PAI-1

PAI: Plasminogen activator inhibitor; TAFI: Thrombin-activatable fibrinolysis inhibitor; FXIII: Factor XIII; Lp(a): Lipoprotein (a).

**Table 2:** Summary of measurements of fibrinolysis, its clinical application, strengths and limitations.

Methods of Measurement of Fibrinolysis	Clinical Application		Strengths and Limitations
	Detection of High Risk Patients	Prediction of Clinical Outcomes	
Electrocardiography (ECG)	Screening ECG does not add value to risk management. Might lead to unnecessary downstream investigations and invasive tests.	ST change resolution does help predict outcomes in patients with ACS	Low cost, low risk. Easily available.
Angiography	Coronary computed tomography angiography (CCTA) screening has been able to identify coronary plaques in asymptomatic individuals.	Well documented evidence of epicardial blood flow on angiogram and correlation to outcomes	Standard test. Invasive and requires use of ionising radiation.
Factorial Assays	Varying reports of correlation between different assays and development of cardiovascular disease	TAFI and PAI 1 have been found to correlate with clinical outcomes.	Fluctuates over time and in response to other clinical factors
Assessment of Clot Structure	Clot structure has been found to be more resistant to lysis in some patients with higher risk.	Limited studies to associate with clinical outcomes	Complex procedure to analyse samples.
Thromboelastography (TEG)	Has shown difference in clot strength (MA) in patients with varying levels of unstable angina	Only clot strength (MA) has been shown to help predict outcome.	Not relevant to arterial thrombosis. Established method in hyperfibrinolytic status, i.e., bleeding. Limited data on hypofibrinolysis. No standard method of using TEG (native or with additives).
The Global Thrombosis Test (GTT)	Clinical studies have shown correlation of impaired lysis with conventional CV risk factors. Impaired lysis is able to detect high risk ACS patients and high risk PPCI patients.	Impaired fibrinolysis shown to be predictive of recurrent adverse cardiovascular events in patients with acute coronary syndrome and renal impairment.	Point of care test. High shear stress which mimics flow within narrowed vessel. Relatively new therefore limited data.

## Abbreviations

ACS	Acute Coronary Syndrome
ADP	Adenosine diphosphate
CAD	Coronary artery disease
CCTA	Coronary computed tomography angiography
CVD	Cardiovascular disease
CLT	Clot lysis time
DIC	Disseminated intravascular coagulation
ECG	Electrocardiogram
ESRD	End-stage renal disease
FXIII	Factor XIII
GRACE	Global Registry of Acute Coronary Event
GTT	Global thrombosis test
Ks	Permeation coefficient/fibrin clot permeability
LDL	Low density lipoprotein
Lp(a)	Lipoprotein (a)
LT	Lysis time
MA	Maximum amplitude
MACE	Major adverse cardiovascular event
OT	Occlusion time
PAI	Plasmin activator inhibitor
PAP	Plasma-α 2-antiplasmin
PCI	Percutaneous coronary intervention
PPCI	Primary percutaneous coronary intervention
ROTEM	Rotational thromboelastometry
STEMI	ST-segment elevation myocardial infarction
TAFI	Thrombin-activatable fibrinolysis inhibitor
TCFA	Thin-cap fibroatheroma
TEG	Thromboelastography
TIMI	Thrombolysis in myocardial infarction
tPA	Tissue plasminogen activator
TxA2	Thromboxane A2
uPA	Urokinase plasminogen activator
vWF	Von Willebrand Factor
